# A New Cluster Analysis-Marker-Controlled Watershed Method for Separating Particles of Granular Soils

**DOI:** 10.3390/ma10101195

**Published:** 2017-10-18

**Authors:** Md Ferdous Alam, Asadul Haque

**Affiliations:** Department of Civil Engineering, Monash University, Melbourne, Victoria 3800, Australia; ferdous.alam@monash.edu

**Keywords:** X-ray CT imaging, granular soils, particle separation, watershed method

## Abstract

An accurate determination of particle-level fabric of granular soils from tomography data requires a maximum correct separation of particles. The popular marker-controlled watershed separation method is widely used to separate particles. However, the watershed method alone is not capable of producing the maximum separation of particles when subjected to boundary stresses leading to crushing of particles. In this paper, a new separation method, named as Monash Particle Separation Method (MPSM), has been introduced. The new method automatically determines the optimal contrast coefficient based on cluster evaluation framework to produce the maximum accurate separation outcomes. Finally, the particles which could not be separated by the optimal contrast coefficient were separated by integrating cuboid markers generated from the clustering by Gaussian mixture models into the routine watershed method. The MPSM was validated on a uniformly graded sand volume subjected to one-dimensional compression loading up to 32 MPa. It was demonstrated that the MPSM is capable of producing the best possible separation of particles required for the fabric analysis.

## 1. Introduction

The separation of images of solid particles of granular soils subjected to boundary stresses is essential for studying the particle-level fabric. The applied boundary stress causes the solid particles to develop inter-particle forces at contacts, which affects the particle-level fabric resulting from sliding, rolling, and crushing of particles. Researchers have been investigating the fabric of granular soils using the most convenient and non-invasive imaging techniques: X-ray computed tomography and synchrotron radiation computed tomography. In situ imaging of granular soil fabric subjected to various loading have been investigated, including triaxial loading [1,2,3,4,5,6,7,8], one-dimensional compression [9,10], and plane-strain (biaxial loading) [3,11,12]. With the advancement of high-resolution computed tomography and image segmentation techniques [13,14,15,16,17], it is now possible to investigate the particle-level fabric [1,2,6,8,9,18,19,20] required for an in-depth understanding of the mechanical response. However, the analysis of particle-level fabric requires separation of particles after segmentation. The importance of accurate separation of particles to study the fabric of granular soils has already drawn the attention of researchers [19]. However, the need for the maximum separation of particles for an accurate analysis of the particle-level fabric of granular soils has not yet gained an appropriate level of attention due to the inherent challenges envisaged in the development of separation algorithms. Inaccurate separation may result in erroneous fabric parameters, which may grossly undermine the benefits of particle-level fabric analysis.

The watershed algorithm [13] has been widely used by many researchers in geotechnical engineering to separate particles [1,2,4,6,8,9,14]. Among all of the available types of watershed algorithms, the marker-controlled approach [15,16] is the most expedient, where the algorithm simulates flooding from the markers and inflates the regions according to the increasing values of a priority map until the regions extend to the watershed lines. In this method, the number of separated particles is the same as the number of labelled markers. One very common approach is to use the distance map (e.g., Chamfer, Euclidean) of particles’ binary volume to be separated as the priority map (landscape height field) and to extract the markers from the regional maxima of the distance map [16,21]. However, the regional maxima alone without any further mathematical treatment usually results in over-separation. The h-maxima transformation of the distance map is a powerful mathematical tool to suppress the undesirable maxima, which has been implemented by several researchers [9,14]. This transformation suppresses all the maxima whose depth is lower than or equal to a given threshold value of *h* [16,21], which has a direct effect on the number of separated particles.

The current practice of selecting an appropriate *h*-value for the effective separation of particles is based on visual examination of the quality of separated particle volumes. However, it becomes almost impossible to find an acceptable *h*-value that ensures the best possible separation of particles with significant variations of size and morphology resulting from the applied boundary stresses [4,13]. Although visual examination is the ultimate factor to make an evaluation [9,12,17], a rigorous mathematical framework is necessary to reach a sufficiently accurate solution with minimal human interference. This paper presents a new cluster analysis-marker-controlled watershed method for separating solid particles of granular soils, named herein as the Monash Particle Separation Method (MPSM). This method consists of an automatic mathematical technique for determining an optimal *h*-value for the maximum accurate separation of particles, and an interactive, semi-automatic clustering by Gaussian mixture models [22,23] for separating the unseparated particles, which cannot be separated otherwise. The detailed formulation of the MPSM by using simple particle configurations is presented, including its validation on a uniformly graded sand volume subjected to one-dimensional compression loading up to 32 MPa. Results of the separation outcomes for all image volumes are presented to demonstrate the noticeable improvements that could be achieved by the MPSM.

## 2. Materials and Methods

### 2.1. Materials

The sand particles used in this study were collected from the basement excavation (depth ≈ 13.0 m from the existing ground level) for the new Learning and Teaching Building at Monash University’s Clayton Campus, Australia. The sands are locally known as Red Bluff Sands and belong to the Brighton Group materials, which are predominantly comprised of quartz minerals with small amounts of iron oxides [24,25]. The sand sample was initially wet-sieved using a 75 µm sieve [26] to discard the fines. The coarse fraction was dried in the oven for a 24-h duration at 105 ± 0.5 °C. For this investigation, the dried sample was sieved through 300 µm and 150 µm sieves [26] to prepare a uniformly graded sand sample (300–150 µm). The specific gravity of the sand particles measured using a Multipycnometer (Quantachrome Instruments, Boynton Beach, FL, USA) was 2.65.

### 2.2. Experimental Setup and Image Acquisition

**Figure 1:**
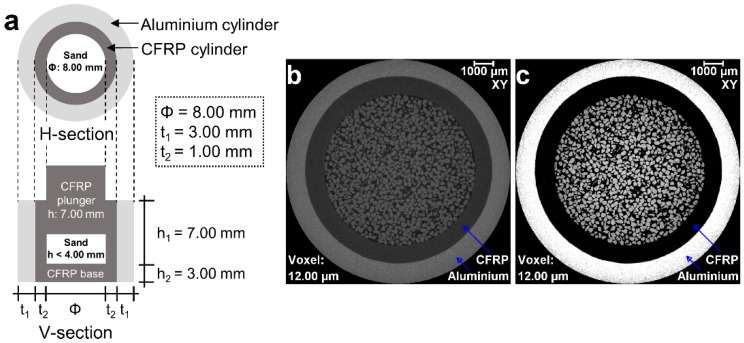
(**a**) Plan view and elevation of the new carbon fibre reinforced polymer (CFRP) compression cell encapsulated in an aluminium cylinder with plunger and base support; (**b**) an orthoslice of the reconstructed sand volume (intensity range: 0 to 65,535); and (**c**) an orthoslice showing the ease of particle segmentation due to inclusion of the CFRP (intensity range: 14,500 to 24,700).

The load-stage selected for this study was one-dimensional compression [27,28], which primarily requires a cell to confine a cylindrical specimen radially for subsequent compression in the direction (*Z*-axis) perpendicular to the diameter. Previously, researchers used an aluminium cylinder due to its low X-ray absorption capacity [9]. However, the segmentation of sand particles at the sand–cylinder interface was difficult due to the poor contrast between sand and aluminium [9]. In this study, a new carbon fibre reinforced polymer (CFRP, a radiolucent material) cell encapsulated in an aluminium cylinder was designed to overcome the contrast issue (Figure 1). Also, a solid CFRP plunger for applying the compressive load and a CFRP base was used (Figure 1a). The ease of segmentation of the sand particles can easily be understood by comparing the output, as shown in Figure 1b (whole intensity range) and Figure 1c (intensity range for sands and outer aluminium cell separated by the carbon fibre reinforced polymer in the middle). This new setup is capable of holding the specimen having a diameter to height ratio of 2 to 3, thus satisfying the standards [27,28].

For the compression test, the sieved sands of 0.3 g were poured into the cell from a height of about 15 mm and lightly tamped horizontally and vertically. The whole setup was then placed onto a 5 kN load-stage, (CT5000, Deben, UK) [29], where the compressive load was applied by the upward movement of the bottom platen. The loading and data acquisition were controlled by the MICROTEST (Version 6.13) software [30] that comes with the load-stage. The sand specimen had an initial dry density of 1600 kg/m^3^ and a void ratio of 0.66. An in situ X-ray image was taken of the initial sample (i.e., no load condition) prior to increasing the compressive stresses to 8, 16 and 32 MPa by slowly moving the bottom platen up at 0.1 mm/min. These stresses were selected from the pre-crushing (0 and 8 MPa) and post-crushing (16 and 32 MPa) stress ranges [9] to simulate varying degrees of difficulties with particle-level separation. For each applied stress, the sand sample was left for about half an hour under the constant stress to reach the deformation reading an asymptotic state. Subsequently, in situ imaging was completed by keeping the platen fixed. A 3D-Zeiss Xradia 520 Versa Microscopy facility (Xradia, Pleasanton, CA, USA) [9], which was established from the funding of the Australian Research Council, was used. For the imaging, a total of 1601 projections with a voxel size of 12 µm and a frame size of 1024 × 1024 pixels were taken by rotating the load-stage 360° around its vertical axis (*Z*-axis). The scan setup comprised of source to specimen distance = 68.57 mm, specimen to detector distance = 322.65 mm, voltage = 140 keV, power = 10 W, exposure = 3 s, camera binning = 2 and lens = 0.4×. 3D volumes were reconstructed from the 2D projections using the XMReconstructor software [31].

### 2.3. Image Post-Processing

Post-processing of the reconstructed images was accomplished using Avizo image processing software [21]. The reconstructed image volumes containing the sand specimen were subjected to 2D version (XY plane) non-local means filter [21,32]. The auto-thresholding module consisting of Entropy [21,33,34], Factorisation [20,21], Moments [21,35] and IsoData [21] methods was used to determine the preliminary intensity threshold values for segmenting the solid sand particles. Threshold values were determined for the region bounded by the sand matrix only (i.e., sands and voids). The final threshold values for segmenting particles from the entire volumes were determined using the interactive thresholding module and human judgement [9,17]. In this process, the calculated mass (0.29 g) of the processed binary volumes of sands was maintained close to the measured mass of 0.30 g. These segmented image volumes were then used for further analysis.

### 2.4. Determination of Optimal h-Maxima Contrast Value

The routine marker-based watershed separation method was applied to the binary volumes with different contrast coefficients (*h*) for the h-maxima transformation using the Chamfer distance map (CDM). The *h*-values selected for the Chamfer distance map were between 0 and 5. The face, edge, and corner distance considered in the Chamfer distance map were 1, 1.414, and 1.732, respectively. The repeatable algorithm mode, which uses the newly computed transformation value of a point as an argument for the untransformed points, and the watershed configuration were selected. In all of the cases, 3D interpretation and 26 neighbourhood (6 faces, 8 corners, and 12 edges) were considered. The resulting separated particles were labelled by considering 26 neighbourhood.

The labelled volume data of the separated particles was exported to MATLAB (Version 2016a,b) [22] as 3D matrix for image analysis. The spatial coordinates of each voxel point (voxel’s centroid) with its label ID were extracted using the voxel size and the origin of the local axes. The coordinates of all voxel points of each particle or group of particles (having a specific label ID) were considered as a clustered data (i.e., the particle label ID is the same as the cluster ID) meaning the number of the particles or groups of particles present in the labelled volume is the same as the number of clusters. The goodness of the clustering (i.e., separation) of each of the labelled volumes was evaluated with two of the most popular cluster evaluation techniques: Calinski–Harabasz criterion [22,36] and Davies–Bouldin criterion [22,37]. The Calinski–Harabasz index value (*CHV*) is defined by Equation (1) [22] as:

(1) $$CHV=\frac{SS_{B}}{SS_{W}}\times \frac{N-k}{k-1}$$

where, *k* is the number of clusters, *N* is the number of observations, *SS_B_* is the overall between-cluster variance and *SS_W_* is the overall within-cluster variance. The between-cluster variance is large and the within-cluster variance is small for well-distinct clusters, resulting in larger *CHV*. So, the larger the *CHV*, the better is the clustering solution.

The overall between-cluster variance is further defined by Equation (2) [22] as:

(2) $$SS_{B}=\sum_{i=1}^{k}n_{i}{\left\|m_{i}-m\right\|}^{2}$$

where, *n_i_* is the number of observations belonging to cluster *i*, *m_i_* is the centroid of cluster *i*, *m* is the overall mean of the sample data, ||*m_i_* − *m*|| is the L^2^ norm (Euclidean distance) between the two vectors and *k* is the number of clusters.

The overall within-cluster variance is defined by Equation (3) [22] as:

(3) $$SS_{W}=\sum_{i=1}^{k}\sum_{x\in c_{i}}{\left\|x-m_{i}\right\|}^{2}$$

where, *x* is a data point, *c_i_* is the *i*th cluster, *m_i_* is the centroid of cluster *i*, ||*x* − *m_i_*|| is the L^2^ norm (Euclidean distance) between the two vectors and *k* is the number of clusters.

The Davies–Bouldin criterion takes into account the ratio of within-cluster and between-cluster distances. The Davies–Bouldin index value (*DBV*) is defined by Equation (4) [22]. The smaller the *DBV* value, the better is the clustering solution.

(4) $$DBV=\frac{1}{k}\sum_{i=1}^{k}\max_{j\neq i}\{D_{i,j}\}$$

where, *D_i,j_* is the within-to-between cluster distance ratio for the *i*th and *j*th clusters and is defined by Equation (5) [22] as:

(5) $$D_{i,j}=\frac{\bar{d_{i}}+\bar{d_{j}}}{d_{i,j}}$$

where, $\bar{d_{i}}$ is the average distance between each point in the *i*th cluster and the centroid of the *i*th cluster; $\bar{d_{j}}$ is the average distance between each point in the *j*th cluster and the centroid of the *j*th cluster; *d_i,j_* is the Euclidean distance between the centroids of the *i*th and *j*th clusters. The minimum value of *D_i,j_* indicates the best-case within-to-between cluster ratio for cluster *i*.

The optimal *h*-value corresponds to the highest *CHV* value or the lowest *DBV* value which produces the maximum possible separation of labelled volumes with the least incorrect separation which could result from the concavity of particles, internal pores etc.

**Figure 2:**
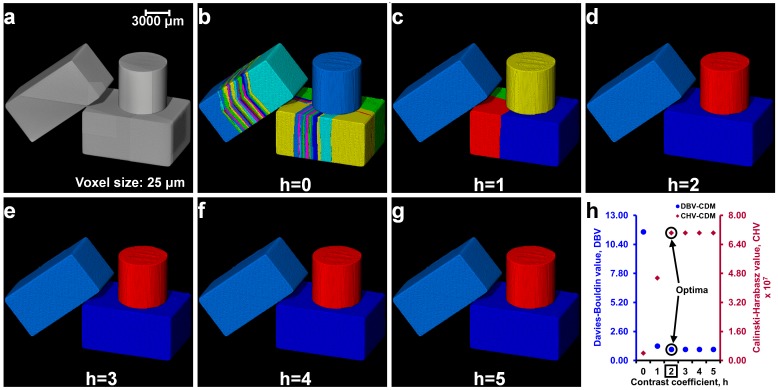
Determination of the optimal contrast coefficient, h, for three regular shaped blocks in contact: (**a**) greyscale volume; (**b**–**g**) separation results (labelled volumes) for different *h*-values (*h* = 0 to 5) with the Chamfer distance map (CDM); and (**h**) cluster evaluation criteria values with the optimal *h*-value (square marked).

The process of separating particles using the optimal *h*-value obtained from the cluster evaluation techniques is the first step of the watershed separation method proposed in this paper (Part I of the MPSM). The separation outcomes of three regular-shaped aluminium blocks (Figure 2a) with micro-roughness at their contacts due to the wire-cut process have been analysed using different *h*-values. Figure 2 shows the separation outcomes of these blocks with *h*-values of 0 to 5. It can be seen that over-separation occurred for *h*-values 0 and 1 (Figure 2b,c), whereas a correct separation was achieved using *h*-value of 2 (Figure 2d). A further increase of *h*-value from 3 to 5 (Figure 2e–g) showed unchanged separation outcomes meaning *h*-values ranging from 2 to 5 were effective to separate all the blocks. The cluster evaluation values for all the *h*-values are presented in Figure 2h. Both the criteria (*DBV* and *CHV*) produced a constant evaluation value for these blocks with the *h*-values of 2 to 5 thus the use of any *h*-value within this range is expected to produce the same separation outcome (Figure 2d–g).

**Figure 3:**
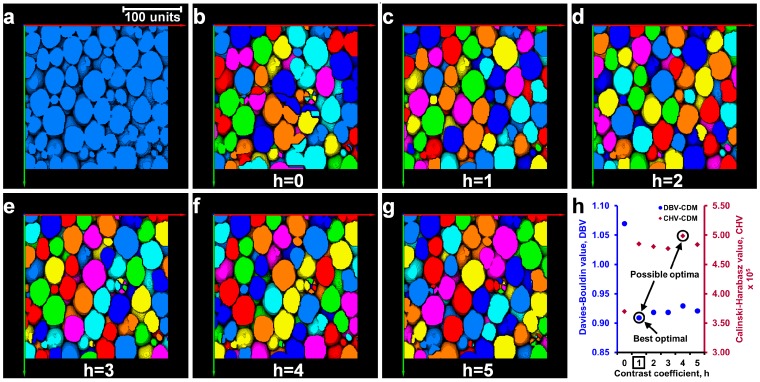
Determination of the optimal contrast coefficient, h, for “foam.am”: (**a**) binary volume; (**b**–**g**) separation results for different *h*-values (*h* = 0 to 5) with the Chamfer distance map (CDM); and (**h**) cluster evaluation criteria values with the optimal *h*-value (square marked).

**Figure 4:**
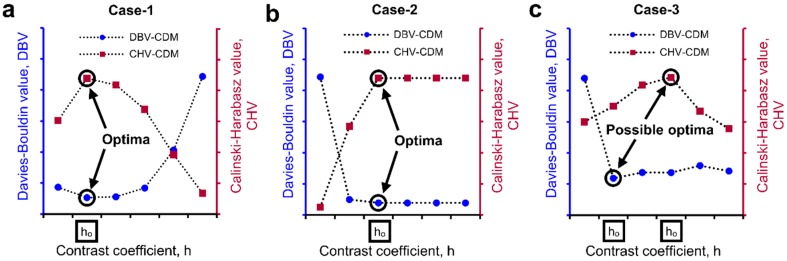
Cluster evaluation values and different cases of optimal *h*-value: (**a**) Case I- one optimal *h*-value; (**b**) Case II- optimal *h*-value over a range; and (**c**) Case III- two possible optimal *h*-values.

Further evaluation of this process has been demonstrated on a relatively complex volume (“foam.am” data was taken from Avizo’s tutorial) [21]. The pores of the foam volume (Figure 3a) were separated using the *h*-values of 0 to 5 (Figure 3b–g). Visual inspection clearly shows that a *h*-value of 1 resulted in a better separation outcome (Figure 3c) while the *h*-values of 0 and 2 to 5 resulted in incorrect (over and/or under) separation of pores (Figure 3b,d–g). In this case, the cluster evaluation criteria yielded two possible optimal *h*-values, *h* = 1 for DBV and *h* = 4 for *CHV* (Figure 3h). When the cluster evaluation criteria produce two different *h*-values, a visual verification of the outcomes obtained from the two possible optimal *h*-values is warranted to confirm the best separation outcome. A *h*-value of 1 based on the *DBV* ensured the best separation of the pores (Figure 3c) without over-separation and is defined as the optimal *h*-value. The integration of such cluster evaluation techniques in selecting an optimal *h*-value from a wide range of *h*-values will reduce the currently-practised human interference significantly. Based on the cluster evaluation exercise demonstrated here, there could be three possible cases of evaluation outcomes: (i) Case I—both cluster evaluation criteria show clearly the same optimal *h*-value (Figure 4a); (ii) Case II—both criteria show a constant cluster evaluation value (Figure 4b) where the lowest *h*-value in the range can be taken as the optimal; and (iii) Case III—both criteria show two different possible optimal *h*-values, which requires human interference to select the best optimal *h*-value (Figure 4c).

The groups of the particles which could not be separated using the marker-controlled watershed method with the optimal *h*-value (Part I of the MPSM) require different separation treatments. In this study, the clustering by Gaussian mixture models has been proposed to separate the remainder unseparated particles (Part II of the MPSM), which would otherwise be unseparated.

### 2.5. Separation of Particles Using Clustering by Gaussian Mixture Models

**Figure 5:**
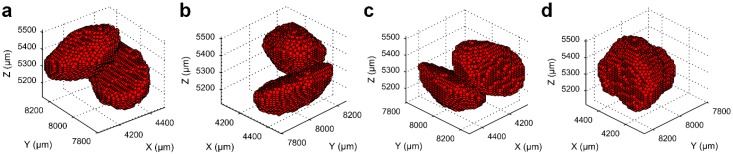
Manual inspection of each particle or group of particles in four different views with automatic and interactive MATLAB program. (**a**) Azimuth = −37.5°, elevation = 30°; (**b**) azimuth = 45°, elevation = 30°; (**c**) azimuth = 135°, elevation = 30°; and (**d**) azimuth = 225°, elevation = 30°.

In order to identify the unseparated particles within the labelled volumes obtained using the optimal *h*-value, an interactive, semi-automatic algorithm was developed in MATLAB. The code generates four 3D simultaneous views of each particle or group of particles one by one (Figure 5) and asks input for the number of unseparated particles, *n* (*n* ≥ 1) based on visual feedback. In the case of already separated particles (*n* = 1), the program calculates the 3D matrix indices for the voxel coordinates and assigns 1 to the matrix elements of those indices. As a result, a 3D matrix of a binary scalar volume of completely separated particles was created. Similarly, another matrix for the unseparated particles (i.e., *n* ≥ 2) was also created. These unseparated particles or groups of particles were separated using the clustering by Gaussian mixture models (GMM) [22,23] based on the number of clusters or particles as an input value.

**Figure 6:**
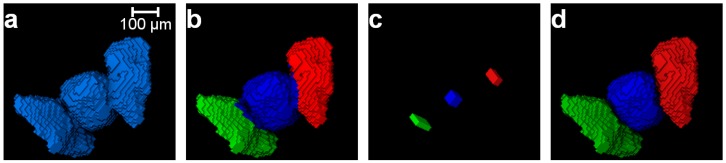
Determination of acceptable marker size for the separation of particles: (**a**) a group of particles; (**b**) cluster analysis; (**c**) extraction of markers; and (**d**) separated particles.

The GMM consists of *n* multivariate normal density components, where *n* is the number of unseparated particles in a group (e.g., *n* = 3 in Figure 6a). For the 3D data, each component has a 3D mean, a 3 × 3 covariance matrix, and a mixing proportion. The mixing proportion of a component is the proportion of the original data that belongs to that component. For fitting the GMM, the Expectation-Maximization (EM) algorithm [22] was used, which requires the initial mean, the covariance matrix, and the mixing proportion (known as the initial conditions). In this investigation, the k-means++ algorithm [22,38] was used to get the initial n clusters with all the initial conditions required for fitting the GMM. Once the initial conditions are known, the EM algorithm then calculates the posterior probabilities of each data point (E-step). For n components, each voxel point will have n posterior probabilities, each of which indicates the probability of that point belonging to a component. Using these posterior probabilities as weights, the algorithm again calculates the component covariance matrices, means, and mixing proportions by applying the maximum likelihood (M-step). Iteration of the EM algorithm continues over these E and M steps until the convergence of the likelihood has reached. In this investigation, a maximum of 1000 iterations (based on several trials to produce satisfactory clustering outcomes within reasonable computational time) was used for each of the clusters. After fitting the GMM to the unseparated particles, each voxel point was assigned exactly to one cluster (hard clustering) which yielded the highest posterior probability.

**Figure 7:**
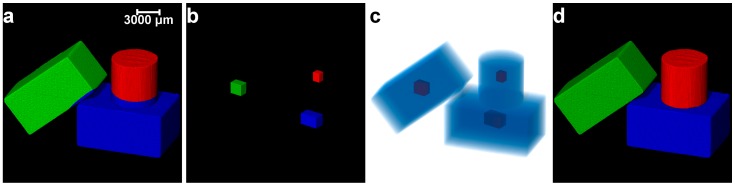
Separation of three blocks using the GMM clustering: (**a**) clusters; (**b**) extracted markers; (**c**) markers imposed on the blocks; and (**d**) separated blocks.

**Figure 8:**
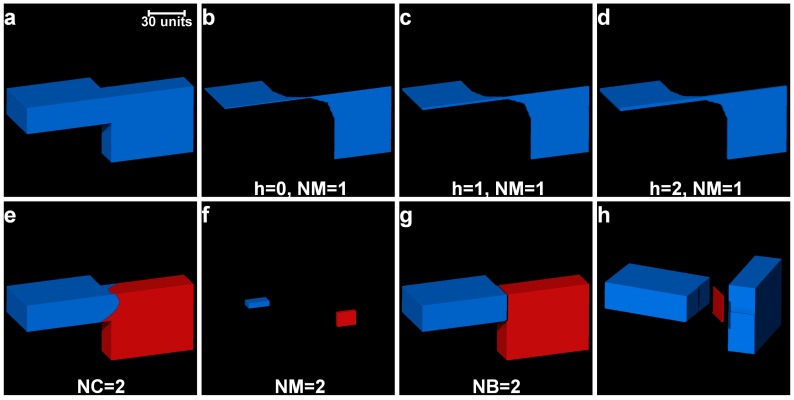
Separation of two virtual blocks having perfect contact using the GMM clustering: (**a**) binary image; (**b**–**d**) markers for *h* = 0–2; (**e**) clusters; (**f**) extracted markers; (**g**) separated blocks; and (**h**) blocks and contact surface. [NM: no. of markers, NC: no. of clusters, NB: no. of blocks].

Once the clusters are determined (e.g., Figure 6b), the principal component analysis [22,39] was performed for each cluster to determine the three principal axes lengths [8]. Using a percentage of the principal axes lengths, a cuboid region of interest (ROI) having the same centroid as that of the cluster was established. The percentage of the principal length defining the size of the ROI was determined by conducting a parametric study on the three unseparated particles (Figure 6a). Table 1 shows the effect of varying percentages of principal axes lengths on the separation outcomes. The length percentages smaller than 8% and greater than 70% produced erroneous separation outcomes (Table 1). In this study, a length percentage of 20% (marked as bold in Table 1) was used to define the cuboid ROI. The voxel points within the cuboid ROI including their spatial coordinates were determined and converted to corresponding 3D matrix indices followed by assigning 1 to the elements of those indices, thus producing a cuboid marker. The 3D matrix containing the cuboid markers was labelled by considering 26 neighbourhood in Avizo (e.g., Figure 6c). Subsequently, the cuboid marker-controlled watershed method, with the exception to the h-maxima transformation, was applied to separate the particles (e.g., Figure 6d). The GMM clustering algorithm was initially applied to the three regular-shaped aluminium blocks with imperfect contacts due to micro-roughness (Figure 7). Although the GMM clustered some of the voxels to the incorrect blocks (Figure 7a), the markers were extracted accurately for all the blocks (Figure 7b,c) and augmented successfully with the marker-controlled watershed algorithm as evidenced from the final separation outcome (Figure 7d). In addition, the GMM clustering was applied to separate two virtual blocks in perfect contacts (Figure 8a). The newly introduced separation method using the cuboid markers extracted from the GMM clustering (Figure 8e,f) was successful in separating these blocks correctly (Figure 8g,h), whereas the classical watershed algorithm was incapable due to the single marker for all *h*-values (Figure 8b–d).

**Figure 9:**
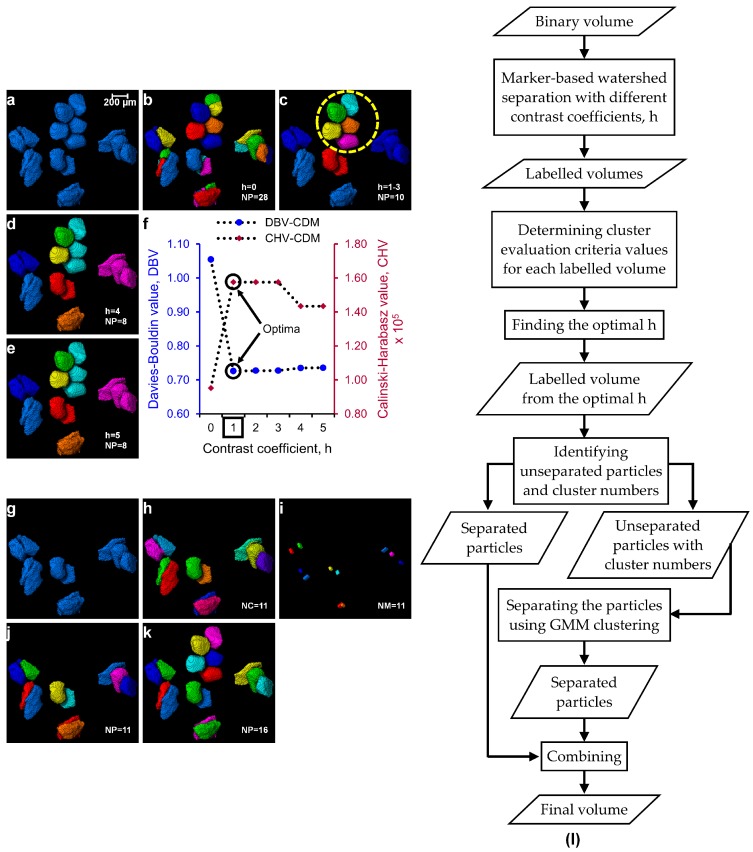
(**a**) Challenging groups of particles; (**b**–**e**) separation outcomes using the watershed method with *h* = 0 to 5; (**f**) optimal *h*-value based on the cluster evaluation values (**g**) remaining unseparated particle-groups; (**h**) GMM clustering; (**i**) generation of cuboid markers; (**j**) separation outcomes for the unseparated groups; (**k**) combined volume with all particles separated; and (**l**) flow chart showing the MPSM. [NP: no. of particles, NC: no. of clusters, NM: no. of markers].

The entire MPSM, including Part I and Part II, has been validated using a complex volume of sand particles containing unseparated particles (Figure 9a). Separation outcomes using different *h*-values (0 to 5) in the watershed framework are shown in Figure 9b–e. It can be seen clearly that a *h*-value smaller than 1 (Figure 9b) and greater than 3 (Figure 9d,e) results in over- and under-separation, respectively. Part I of the MPSM determines the optimal *h*-values of 1 to 3 based on the two cluster evaluation criteria, *DBV* and *CHV* (Figure 9f). In this study, the minimum *h*-value of 1 is considered as the optimal *h*-value, which produces the best separation outcome without over-separation (Figure 9c). Moreover, no *h*-value including the optimal *h*-value was able to separate the five particle-groups containing two or more particles in each group except those particles within the marked circle (Figure 9c). The unseparated particle-groups were separated using Part II of the MPSM (Figure 9g–j). The GMM produced 11 clusters (Figure 9h) with 11 cuboid markers (Figure 9i) for the 5 groups of particles. The resultant cuboid markers were used as an input to the marker-controlled watershed method to separate all five particle-groups to 11 single particles (Figure 9j). Finally, the two volumes generated from Part I and Part II of the MPSM were added together to produce the best separation outcome (Figure 9k). A flowchart depicting the entire MPSM separation process is shown in Figure 9l.

The MPSM was applied to separate solid particles of a uniformly graded sand volume subjected to one-dimensional compression loading of 0, 8, 16, and 32 MPa. The sand volumes compressed under 16 and 32 MPa experienced particle crushing and are relatively challenging to separate compared to other two volumes (0 and 8 MPa). The separation outcomes of all the volumes using the routine watershed separation and the MPSM are presented in the subsequent sections.

## 3. Results and Discussion

### 3.1. Separation of Particles Using the Routine Watershed Method

**Figure 10:**
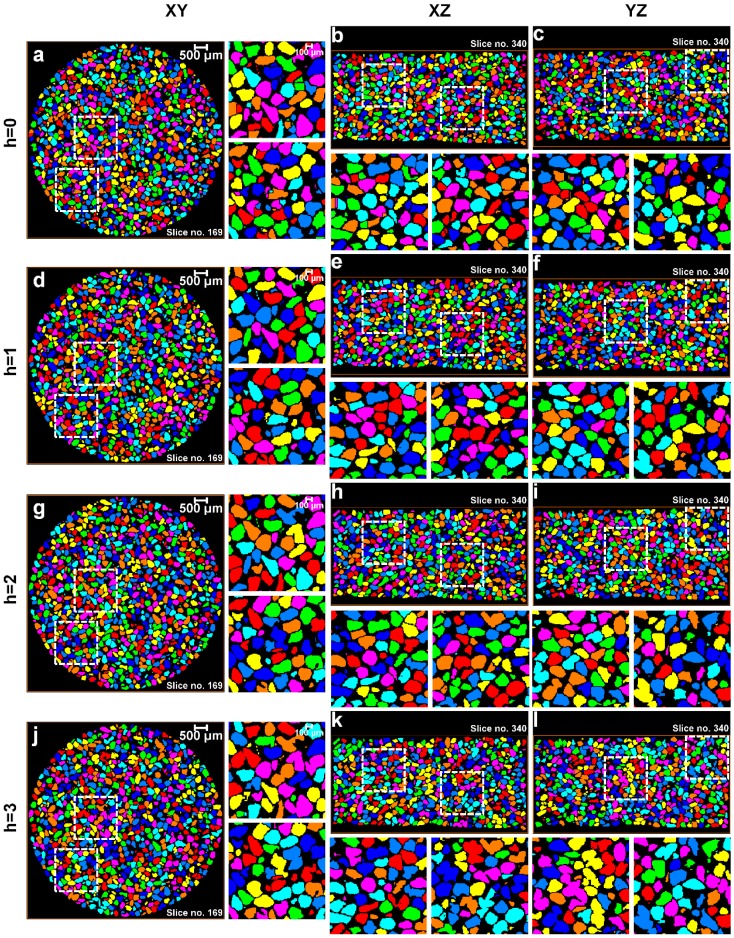
Separation outcomes (XY, XZ, and YZ views through the centre of the sand volumes) for the sand volume compressed under no load and separated using the watershed method with a *h*-value of: (**a**–**c**) *h* = 0; (**d**–**f**) *h* = 1; (**g**–**i**) *h* = 2; and (**j**–**l**) *h* = 3.

**Figure 11:**
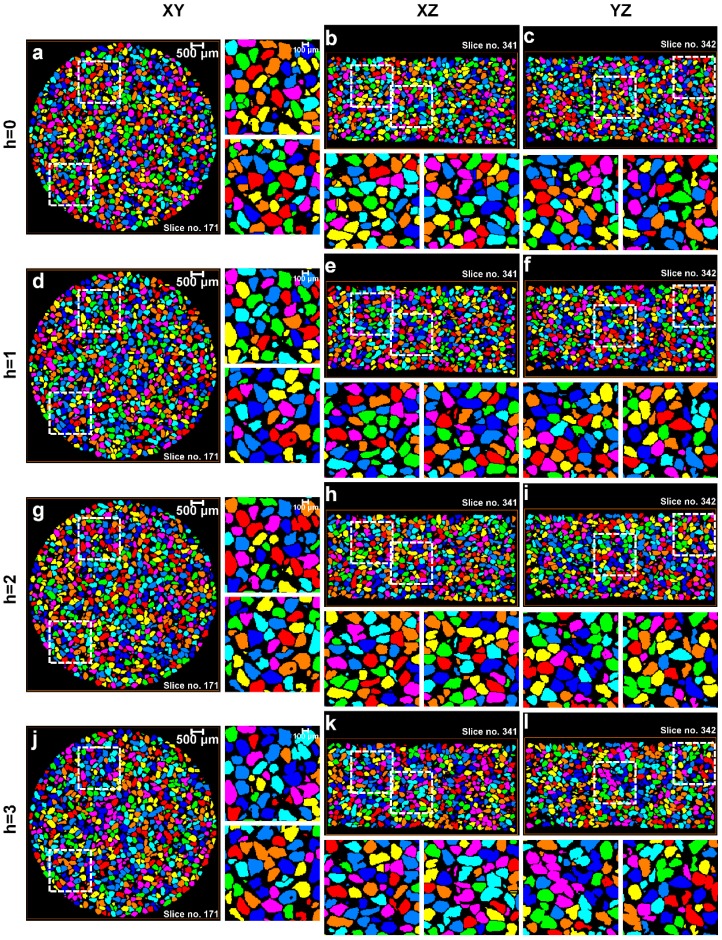
Separation outcomes (XY, XZ, and YZ views through the centre of the sand volumes) for the sand volume compressed under 8 MPa stress and separated using the watershed method with a *h*-value of: (**a**–**c**) *h* = 0; (**d**–**f**) *h* = 1; (**g**–**i**) *h* = 2; and (**j**–**l**) *h* = 3.

**Figure 12:**
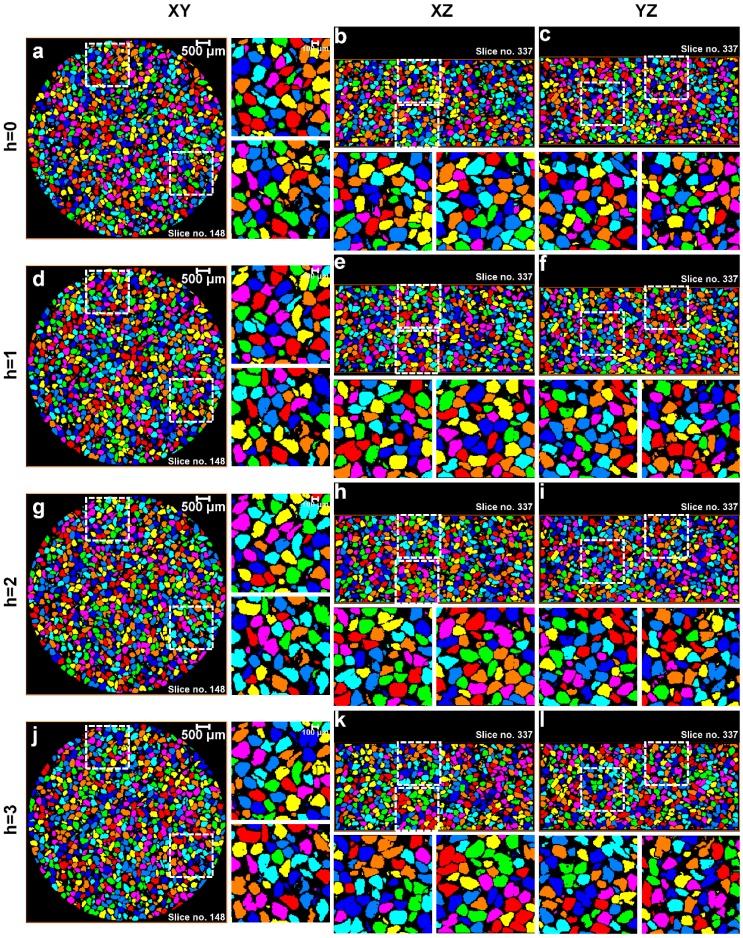
Separation outcomes (XY, XZ, and YZ views through the centre of the sand volumes) for the sand volume compressed under 16 MPa stress and separated using the watershed method with a *h*-value of: (**a**–**c**) *h* = 0; (**d**–**f**) *h* = 1; (**g**–**i**) *h* = 2; and (**j**–**l**) *h* = 3.

**Figure 13:**
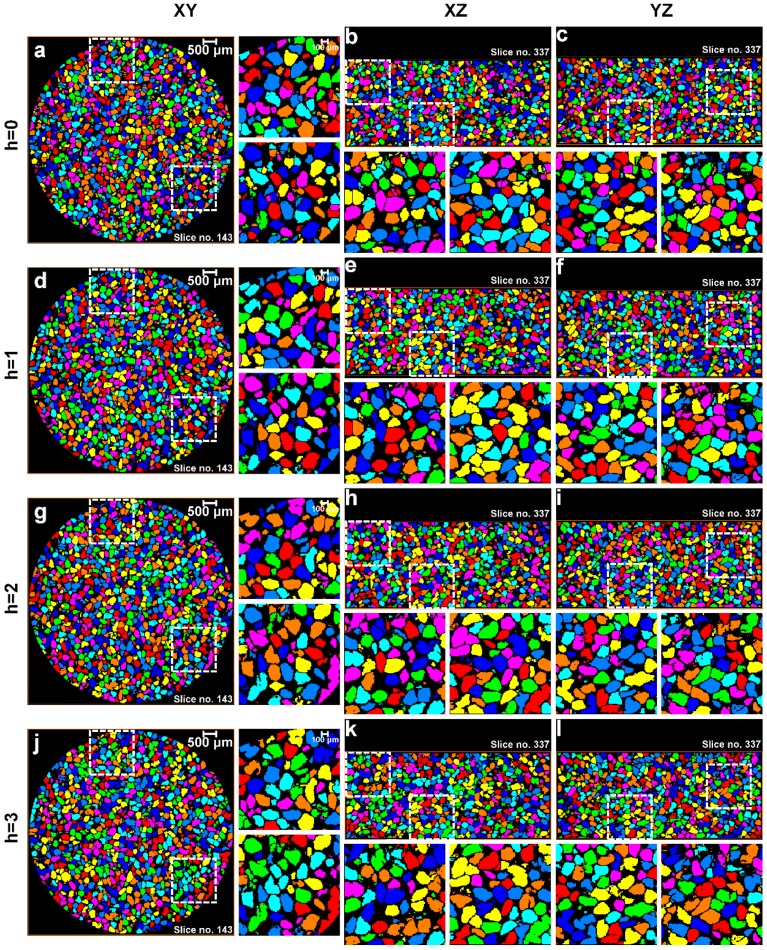
Separation outcomes (XY, XZ, and YZ views through the centre of the sand volumes) for the sand volume compressed under 32 MPa stress and separated using the watershed method with a *h*-value of: (**a**–**c**) *h* = 0; (**d**–**f**) *h* = 1; (**g**–**i**) *h* = 2; and (**j**–**l**) *h* = 3.

All segmented volumes of the sand particles were subjected to routine watershed separation with *h*-values ranging from 0 to 5. The XY, XZ, and YZ view through the center of the labelled particle volumes for 0, 8, 16, and 32 MPa up to a *h*-value of 3 are shown in Figure 10, Figure 11, Figure 12 and Figure 13, respectively. For each volume and plane, two additional enlarged views (right side of the XY and bottom of the XZ and YZ sections, highlighted with square ROIs) are presented for routinely used visual examination of the quality of the separation outcomes.

For the initial volume (Figure 10) under no load condition, it can be seen that *h* = 0 over-separates particles (Figure 10a–c), *h* = 1 provides the best possible separation outcome (Figure 10d–f), and *h* > 1 under-separates particles (Figure 10g–l). Figure 11 presents the volume for 8 MPa, *h* = 0 again over-separates (Figure 11a–c), *h* = 1 produces the best outcome (Figure 11d–f), and *h* > 1 results in under-separation (Figure 11g–l). Similar to the no load and 8 MPa volumes, the volumes compressed under 16 MPa (Figure 12) and 32 MPa (Figure 13) stresses clearly show the best possible separation outcome with *h* = 1.

Surprisingly, the *h*-value of 1 was reported by al Mahbub and Haque [9] for separating the sand particles using the routine watershed separation through visual inspections. This process may become cumbersome and produce an erroneous outcome when a large number of particles are to be separated. Moreover, as the particles are subjected to stress exceeding the crushing stress, smaller particles, unlike the volumes in the pre-crushing region, are expected to evolve due to crushing. A closer examination of all the particle volumes separated using the routine watershed method with the optimal *h*-value (*h* = 1) shows a significant rise in the number of unseparated particle groups with increased compression, particularly from 16 to 32 MPa (Figure 12 and Figure 13). Therefore, the quality of the separation outcomes from the routine watershed method using different *h*-values alone cannot be fully confirmed unless some mathematical framework has been employed to support the claim, which is the focus of the new MPSM.

### 3.2. Separation of Particles Using the MPSM

**Figure 14:**
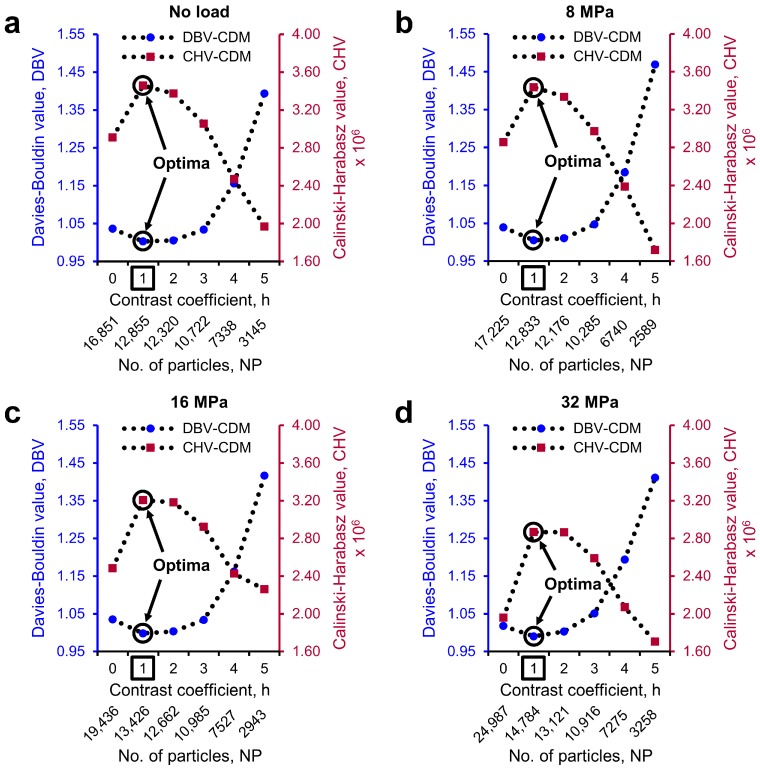
Variations of cluster evaluation values with the contrast coefficients (*h*-values) of 0 to 5 for the sand volume subjected to one-dimensional compression loading of: (**a**) no load; (**b**) 8 MPa; (**c**) 16 MPa; and (**d**) 32 MPa. Circle marks on the plots and square marks on the *X*-axes show the optimal conditions.

Part I of the MPSM evaluates the *h*-value against the best possible separation quality using the two cluster evaluation techniques (*DBV* and *CHV*). Figure 14 shows the variations of *DBV* and *CHV* for all the sand volumes (Figure 14a for no load, Figure 14b for 8 MPa, Figure 14c for 16 MPa, and Figure 14d for 32 MPa) processed with different *h*-values (0 to 5) and the Chamfer distance map. For all these volumes, a minimum of the *DBV* and a maximum of the *CHV* requiring for the best possible separation outcome was obtained at *h* = 1 (marked with black circles on the plots). This coincides with the previously obtained optimal value of *h* = 1 through visual examinations (Section 3.1). Therefore, the proposed method of determining the optimal *h*-value will add significant value to the currently used watershed particle separation method, where a user needs to input a *h*-value and judge the quality of the outcomes of many volumes through subjective visual examinations.

**Figure 15:**
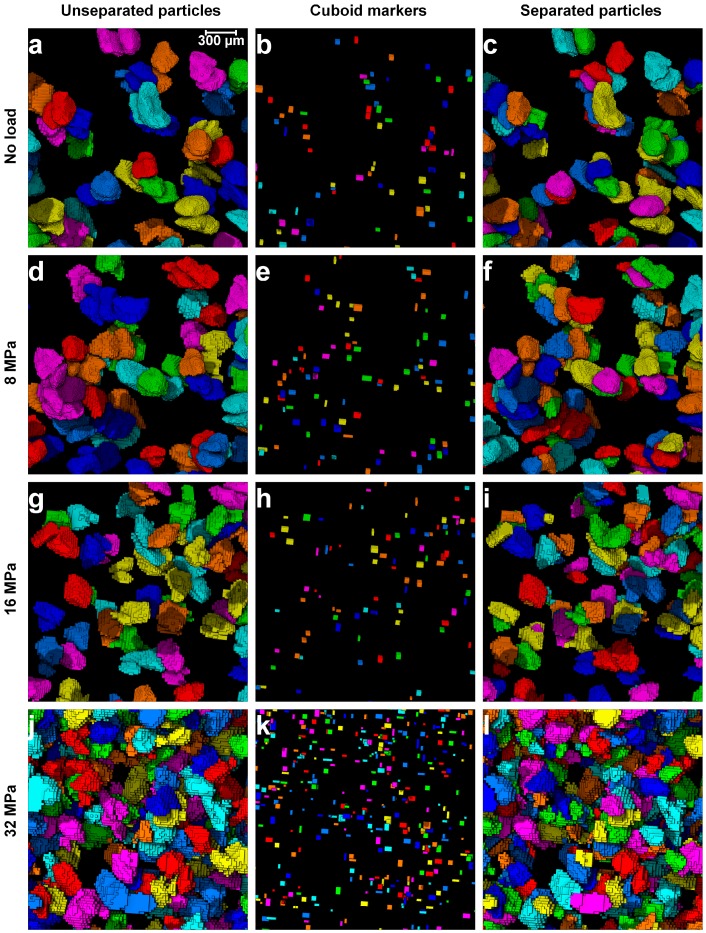
Close-up views of the unseparated particles remaining at the end of the watershed separation with the optimal *h*-value, cuboid markers and separated particles using the Gaussian mixture models for the sand volume compressed under: (**a**–**c**) no load; (**d**–**f**) 8 MPa; (**g**–**i**) 16 MPa; and (**j**–**l**) 32 MPa.

Part II (GMM clustering) of the MPSM was applied to the remaining unseparated particle groups (147 under no load, 183 under 8 MPa, 290 under 16 MPa, and 860 under 32 MPa) which could not be separated by Part I of the MPSM or the routine watershed method. Figure 15 shows close-up views of some remaining unseparated groups of particles under 0, 8, 16, and 32 MPa stresses. As expected, the number of remaining unseparated particle groups increased significantly with the increase of stress (Figure 15a,d,g,j). This indicates that the use of separation outcomes from Part I of the MPSM or the routine watershed method could produce an inaccurate particle-level fabric parameters of granular soils. The GMM featuring Part II of the MPSM enabled the extraction of valuable cuboid markers (Figure 15b,e,h,k), which was finally integrated into the watershed method to separate the challenging unseparated particle-groups to individual particles (Figure 15c,f,i,l). This resulted in 295 individual particles from 147 groups under no load, 369 individual particles from 183 groups under 8 MPa, 587 individual particles from 290 groups under 16 MPa and 1777 individual particles from 860 unseparated groups. The resultant separated particle volumes obtained from the new MPSM is expected to make a significant impact on the analysis of fabric of granular soils.

### 3.3. Particle Size Distributions

**Figure 16:**
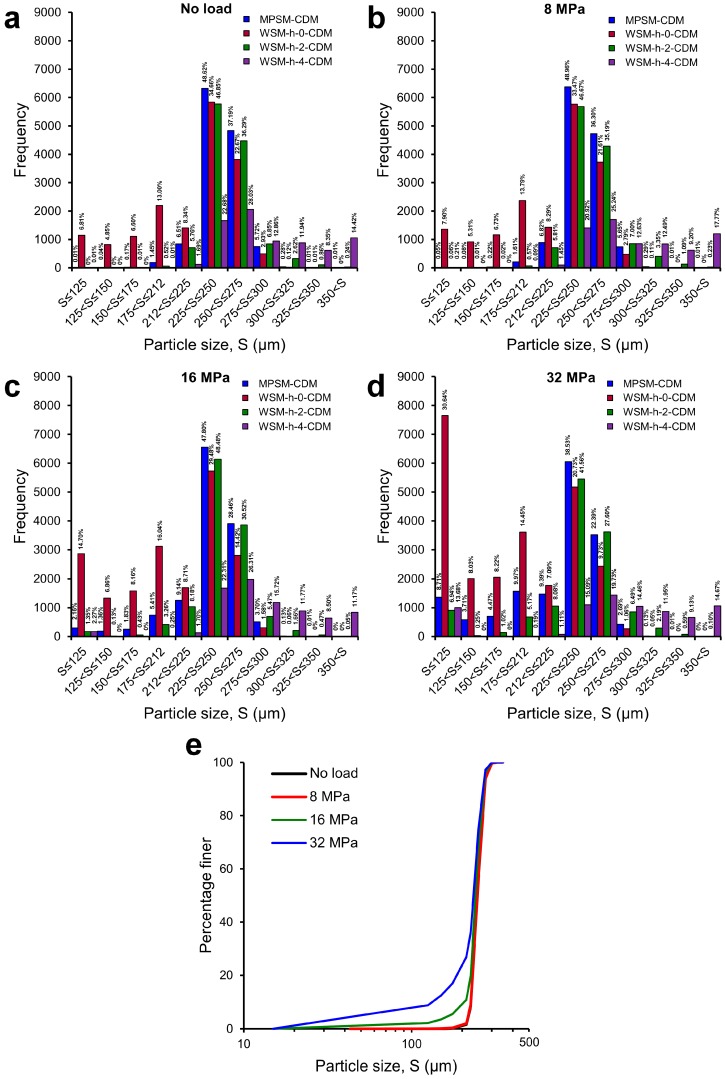
Particle size frequency distributions based on the watershed separation method and the MPSM for the sand volume subjected to one-dimensional compression of: (**a**) no load; (**b**) 8 MPa; (**c**) 16 MPa; (**d**) 32 MPa; and (**e**) size distributions for all loads.

The effects of the routine watershed method on the particle size (as equivalent sphere diameter) frequency plots of the sand volume subjected to 0, 8, 16, and 32 MPa compressive stresses and separated using the MPSM and the routine watershed method with *h*-values of 0, 2, and 4 are shown in Figure 16. As observed before, a *h* = 0 resulted in a significant increase of fine particles as a result of over-separation of particles even under the stress well below the crushing stress (Figure 16a,b). With the increase of stress to post-crushing values (16 and 32 MPa), the percentages of fine particles (<125 μm) markedly increased from a low value of 7% in the pre-crushing range to 14% under 16 MPa (Figure 16c) and 30% under 32 MPa (Figure 16d). The significantly high percentage of fine particles were generated as a result of over-separation of particles as observed previously (Figure 10, Figure 11, Figure 12 and Figure 13). It shows that a *h*-value larger (*h* = 2 and 4) than the optimal *h*-value (*h* = 1) resulted in under-separation of particles thus leading to increased percentages of large particle sizes that were absent in the initial volume (>300 μm).

Particle size distributions using the MPSM with the optimal *h*-value and GMM clustering are also presented in Figure 16. The distribution shows some interesting outcomes, particularly for the two dominant particle size ranges (225–250 μm and 250–275 μm). The MPSM distributions clearly separated particles to the best possible numbers with about 48% particles belong to size 225–250 μm and 37% belong to size 250–275 μm under 0 and 8 MPa stresses (Figure 16a,b), respectively. Immediately after the crushing stress (i.e., 14 MPa) [9], although the 225–250 μm size particle distribution remained unchanged (48%), the number of larger particles (250–275 μm) reduced to about 28% (Figure 16c). This has been correctly reflected by the increase of fine particles (<225 μm). Further increase of stress to 32 MPa shows a considerable drop of particle distribution of size 225–250 μm from 48% to 38% and size 250–275 μm from 28% to 22% (Figure 16d), which is a clear demonstration of the evolution of particle size distributions under increasing stresses (Figure 16e). One of the significant outcomes to note here is that an insignificant number of particles of size greater than the maximum size of particles (i.e., 300 μm) was present in the distributions obtained from the MPSM, which could not be ensured previously [9]. This insignificant number of particles has a very negligible effect on the overall particle size distributions, as shown in Figure 16e.

## 4. Conclusions

In this paper, a new cluster analysis-marker-controlled watershed method (named as the Monash Particle Separation Method) to separate particles of granular soils has been presented. The proposed method automatically determines the optimal *h*-value necessary for the best possible separation of particles using the routine marker-controlled watershed method. The particles, which could not be separated using the optimal *h*-value, were separated using a semi-automated clustering process by Gaussian mixture models producing innovative cuboid markers for the watershed framework. The algorithms developed in this study were implemented initially to assess less complex images prior to its application to relatively complex volumes of uniformly graded granular soils subjected to one-dimensional compression loading up to 32 MPa, which included stresses from pre- and post-crushing stress ranges. It has been successfully demonstrated that the MPSM is capable of maximising the separation of particles present in a granular matrix with the least possible unavoidable inaccurate separation. The separation outcome from the MPSM will have a significant impact on the determination of the particle-level fabric parameters describing the evolution of the microstructure of granular soils. It is envisaged that the implementation of the MPSM in image processing would ensure the best possible separation outcome of granular particles.

## Figures and Tables

**Table 1:** Effects of the percentage of principal lengths on cuboid markers.

Percentage of Length	No. of Markers	No. of Separated Particles (No. of Wrong Separations)	Percentage of Length	No. of Markers	No. of Separated Particles (No. of Wrong Separations)
1	0	0	15	3	3
2	0	0	17.5	3	3
3	2	2	**20**	**3**	**3**
4	3	3 (1)	25	3	3
5	3	3 (1)	30	3	3
6	3	3 (1)	35	3	3
7	3	3 (1)	40	3	3
8	3	3	50	3	3
9	3	3	60	3	3
10	3	3	70	3	3
12.5	3	3	80	1	1
